# A CTC-Cluster-Specific Signature Derived from OMICS Analysis of Patient-Derived Xenograft Tumors Predicts Outcomes in Basal-Like Breast Cancer

**DOI:** 10.3390/jcm8111772

**Published:** 2019-10-24

**Authors:** Hariprasad Thangavel, Carmine De Angelis, Suhas Vasaikar, Raksha Bhat, Mohit Kumar Jolly, Chandandeep Nagi, Chad J. Creighton, Fengju Chen, Lacey E. Dobrolecki, Jason T. George, Tanya Kumar, Noor Mazin Abdulkareem, Sufeng Mao, Agostina Nardone, Mothaffar Rimawi, C. Kent Osborne, Michael T. Lewis, Herbert Levine, Bing Zhang, Rachel Schiff, Mario Giuliano, Meghana V. Trivedi

**Affiliations:** 1 Department of Pharmacy Practice and Translational Research, University of Houston College of Pharmacy, Houston, TX 77204, USA; 2 Lester and Sue Smith Breast Center, Baylor College of Medicine, Houston, TX 77030, USA; 3 Department of Clinical Medicine and Surgery, University of Naples Federico II, 80131 Naples, Italy; 4 Department of Translational Molecular Pathology, MD Anderson Cancer Research Center, Houston, TX 77030, USA; 5 Centre for BioSystems Science and Engineering, Indian Institute of Science, Bangalore 560012, India; 6 Center for Theoretical Biological Physics, Rice University, Houston, TX 77005, USA; 7 Dan L. Duncan Comprehensive Cancer Center, Baylor College of Medicine, Houston, TX 77030, USA; 8 Department of Medicine, Baylor College of Medicine, Houston, TX 77030, USA; 9 Department of Pharmacological and Pharmaceutical Sciences, University of Houston College of Pharmacy, Houston, TX 77204, USA; 10 Center for Functional Cancer Epigenetics, Dana Farber Cancer Institute, Boston, MA 02215, USA; 11 Department of Molecular and Cellular Biology, Baylor College of Medicine, Houston, TX 77030, USA; 12 Department of Bioengineering, Northeastern University, Boston, MA 02120, USA

**Keywords:** circulating tumor cells, CTC clusters, triple-negative breast cancer, patient-derived xenograft, RPPA, transcriptomics, B-cell lymphoma 2, apoptosis

## Abstract

Circulating tumor cell clusters (CTCcl) have a higher metastatic potential compared to single CTCs and predict long-term outcomes in breast cancer (BC) patients. Because of the rarity of CTCcls, molecular characterization of primary tumors that give rise to CTCcl hold significant promise for better diagnosis and target discovery to combat metastatic BC. In our study, we utilized the reverse-phase protein array (RPPA) and transcriptomic (RNA-Seq) data of 10 triple-negative BC patient-derived xenograft (TNBC PDX) transplantable models with CTCs and evaluated expression of upregulated candidate protein Bcl2 (B-cell lymphoma 2) by immunohistochemistry (IHC). The sample-set consisted of six CTCcl-negative (CTCcl−) and four CTCcl-positive (CTCcl+) models. We analyzed the RPPA and transcriptomic profiles of CTCcl− and CTCcl+ TNBC PDX models. In addition, we derived a CTCcl-specific gene signature for testing if it predicted outcomes using a publicly available dataset from 360 patients with basal-like BC. The RPPA analysis of CTCcl+ vs. CTCcl− TNBC PDX tumors revealed elevated expression of Bcl2 (false discovery rate (FDR) < 0.0001, fold change (FC) = 3.5) and reduced acetyl coenzyme A carboxylase-1 (ACC1) (FDR = 0.0005, FC = 0.3) in CTCcl+ compared to CTCcl− tumors. Genome-wide transcriptomic analysis of CTCcl+ vs. CTCcl− tumors revealed 549 differentially expressed genes associated with the presence of CTCcls. Apoptosis was one of the significantly downregulated pathways (normalized enrichment score (NES) = −1.69; FDR < 0.05) in TNBC PDX tumors associated with CTCcl positivity. Two out of four CTCcl+ TNBC PDX primary tumors had high Bcl2 expression by IHC (H-score > 34); whereas, only one of six CTCcl− TNBC PDX primary tumors met this criterion. Evaluation of epithelial-mesenchymal transition (EMT)-specific signature did not show significant differences between CTCcl+ and CTCcl− tumors. However, a gene signature associated with the presence of CTCcls in TNBC PDX models was associated with worse relapse-free survival in the publicly available dataset from 360 patients with basal-like BC. In summary, we identified the multigene signature of primary PDX tumors associated with the presence of CTCcls. Evaluation of additional TNBC PDX models and patients can further illuminate cellular and molecular pathways facilitating CTCcl formation.

## 1. Introduction

The majority of breast cancer (BC)-related mortality is caused by metastasis in women worldwide [1,2]. A key metastatic process involves the dissemination of tumor cells from the primary site to distant organs via the bloodstream [3,4]. The number of circulating tumor cells (CTCs) in the blood is an independent predictor of worse survival in BC patients [5,6,7]. In addition, CTC clusters (CTCcls) represent a unique subset with a better cell survival advantage, higher metastatic potential, and resistance to chemotherapy [8,9,10]. The incidence of CTCcls in metastatic BC (MBC) patients ranges between 17.3% and 40.7% based on different studies conducted with varying sample sizes and CTC detection platforms [9,11,12,13,14]. Interestingly, the median CTCcl counts are reported to be higher in patients with early-stage/locally-advanced BC compared to those with metastatic disease [15]. The clinical prognostic value of CTCcls at baseline and after treatment to predict progression-free survival and overall survival has also been demonstrated in recent studies [16,17,18,19,20,21]. While recent technological advancements have allowed detection and characterization of CTCcls, much remains unknown about the pathways regulating their formation [22].

Irrespective of the investigational platforms employed, many studies, including our own, have detected CTCcls of varying size (2–100 tumor cells/CTCcl) within the circulation of BC animal models and patients [8,11,21,23,24,25,26]. Two possible mechanisms leading to CTCcls formation are either collective migration of tumor cells from the primary site into the circulation or intravascular aggregation of isolated CTCs within the circulation. In support of collective migration, Plakoglobin (also known as γ-catenin), a member of the catenin protein family and also an adaptor protein found in both desmosomes and adherens junction, was demonstrated in a preclinical study [8] to play a crucial role in CTCcl formation and subsequent BC metastasis. This study also reported that the CTCcls were formed from oligoclonal clumps of primary breast tumor cells, using mouse xenograft models [8]. Although the aggregation of CTCs in major blood vessels is unlikely due to the shear stress in circulating blood and a huge excess of hematopoietic cells in the blood in relation to CTCs [9], aggregation of CTCs after intravasation in microvessels surrounding the tumor has been demonstrated recently [27]. Using tissue sections from MBC patients and triple-negative BC patient-derived xenograft (TNBC PDX) models, this study revealed the ubiquitous expression of CD44 (a marker for cancer stem cells [28,29,30,31] and a prognostic predictor in multiple cancer [32,33,34,35,36,37]) in CTCcls compared to single CTCs, and reported the interaction between CD44 and its downstream target p-21 activated kinase 2 (PAK2) as a key mechanism found to mediate tumor cell aggregation [27]. More than one pathway may function independently or simultaneously to dictate the formation and survival of CTCcls. The discovery of additional ‘druggable’ targets to halt CTCcls is important to arrest metastatic progression.

In our previous research on CTCs and CTCcls, we used BC PDX as a model system [24,38]. The BC PDX models serve as the renewable source of CTCs and CTCcls by retaining the genomic, transcriptomic, and biomarker profiles of the original patient-derived tumors that would allow us to better understand their role in metastasis. In this study, our aim was to identify tumor-specific pathways predicting the presence of CTCcls using TNBC PDX transplantable models. We found a higher expression of B-cell lymphoma 2 (Bcl2) and reduced expression of acetyl coenzyme A carboxylase-1 (ACC1) in tumors associated with CTCcl positivity in reverse-phase protein array (RPPA) analysis. In support of this, apoptosis was one of the significantly downregulated pathways, associated with the presence of CTCcls, in the genome-wide transcriptomic analysis. A higher proportion of CTCcl+ tumors than CTCcl− tumors were positive for Bcl2. A CTCcl+ gene signature consisting of 54 upregulated genes was significantly associated with poor relapse-free survival (RFS) in a publicly available dataset from 360 patients with basal-like BC.

## 2. Methods

### 2.1. Selection of BC PDX Models

**Figure 1:**
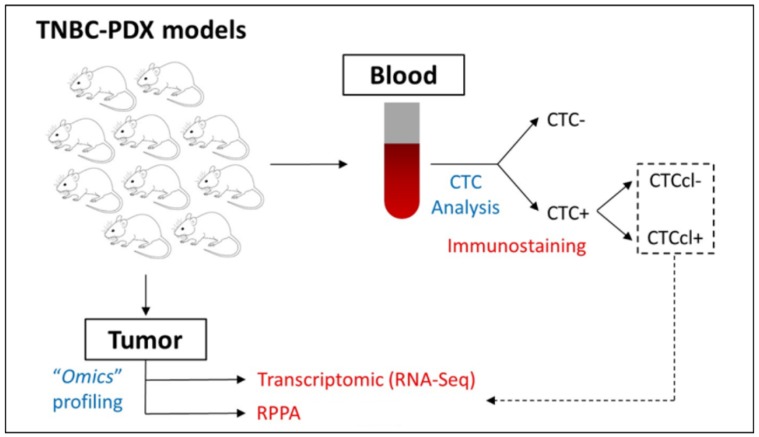
Outline of experimental design. Workflow showing detection of circulating tumor cells (CTCs) and CTC clusters (CTCcls) from patient-derived xenograft (PDX) blood using immunofluorescence staining. The reverse-phase protein array (RPPA) and transcriptomic data from PDX tumors with versus without CTCcls were interrogated and analyzed to identify differentially expressed proteins and genes.

The BC PDX mouse models in our study have been described before [24,39] and have been characterized for the presence of CTCs and CTCcls [24,38]. CTCcls were defined as aggregates of two or more pan-cytokeratin+ CTCs identified by immunohistochemistry (IHC). A total of 10 TNBC PDX models (BCM-3107, BCM-3204, BCM-3561, BCM-3887, BCM-4272, BCM-4664, BCM-5156, BCM-5471, BCM-5998, BCM-6257) were available for the OMICS (RPPA and transcriptomic) analysis described in this study (Figure 1). Out of these PDX models, four (BCM-3204, BCM-3887, BCM-5471, BCM-6257) were CTCcl-positive (CTCcl+), and the rest were CTCcl-negative (CTCcl−) [24].

### 2.2. Protein Analysis by Reverse-Phase Protein Array

Reverse-phase protein arrays analysis was performed as described previously [39]. The RPPA panel included 171 validated antibodies that detect various proteins and phosphoproteins involved in diverse cellular processes ranging from growth factor receptors, nuclear receptors/transcriptional regulators, metabolomic enzymes, and proteins involved in epithelial-mesenchymal transition (EMT), DNA-damage, apoptosis, proliferation, and cell cycle [40]. RPPA quantitative data were available for 6 CTCcl− (BCM-3107, BCM-3561, BCM-4272, BCM-4664, BCM-5156, and BCM-5998) and 3 CTCcl+ (BCM-3204, BCM-3887, and BCM-5471) TNBC PDX models. A false discovery rate (FDR) adjusted *p*-value (*q*-value) threshold of 0.05 was used to define differentially expressed proteins between CTCcl+ and CTCcl− TNBC PDX groups.

### 2.3. Bcl2 Immunohistochemistry

Tumor tissues from available formalin-fixed paraffin-embedded sections of primary PDX tumors were retrieved from the archives. Specimens were used for immunohistochemical staining. Anti-Bcl2 (Mouse monoclonal antibody, clone 124, #M0887) antibody was purchased from Dako, Carpinteria, CA, USA. Immunohistochemistry (IHC) was conducted by processing sections for antigen retrieval (microwaved in 10 mM citrate buffer, pH 6 for 10 min), followed by treatment with 3% H_2_O_2_ in methanol for 10 min and washing in phosphate-buffered saline (PBS) twice. After blocking in 3% bovine serum albumin and 5% goat serum in PBS for 2 h at room temperature, sections were incubated with primary antibodies overnight at 4 °C. Then, sections were washed in PBS and incubated with EnVision-labeled polymer reagent (DAKO, Carpinteria, CA, USA) for 30 min at room temperature. Finally, sections were exposed with nickel, cobalt-3,3-diaminobenzidine (Immunopure Metal Enhanced DAB Substrate Kit; Pierce, Rockford, IL, USA) and counterstained with hematoxylin.

### 2.4. Immunohistochemical Scoring

Protein expression levels were scored by a clinical pathologist blinded to CTCcl status, using a weighted histoscore method, also known as the H-score [41,42,43], which is based on staining intensity (0 (absent), 1+ (weak), 2+ (moderate), or 3+ (strong)) and the percentage of cells stained with that intensity for the full tissue section. The H-score was calculated using the formula (1*(% cells 1+) + 2*(% cells 2+) + 3*(% cells 3+)) that provided a semiquantitative classification of staining intensity, with a maximum score of 300 (if 100% of cells stained strongly positive) and a minimum score of 0 (if 100% of cells were negative). A total of three independent tumors per PDX model were used to assess the histoscore and then averaged to assign the final H-score for each of the 10 TNBC PDX models.

### 2.5. Gene Expression and Network Analysis

RNA-Sequencing (RNA-Seq) data of individual PDX models were retrieved from Gene Expression Omnibus (GEO), a public functional genomics data repository (accession number GSE97726) [44]. Raw fragments per kilobase of transcript per million mapped read values (FPKM) were used for downstream analysis. The sample-set consisted of 6 CTCcl− (BCM-3107, BCM-3561, BCM-4272, BCM-4664, BCM-5156, BCM-5998) and 4 CTCcl+ (BCM-3204, BCM-3887, BCM-5471, BCM-6257) TNBC PDX models. Data were normalized by trimmed mean of M values (TMM) implemented in edgeR (Bioconductor v3.7) and then transformed by voom in limma. For differential expression, genes expressed differentially between the CTCcl+ and CTCcl− were identified using limma (Bioconductor v3.7). A *p*-value < 0.05 was used to define differentially expressed genes between CTCcl+ and CTCcl− TNBC PDX groups. Statistical analysis was performed by a moderated t-test implemented in limma. Wikipathway database was used for pathway enrichment [45], while enrichment analysis was performed by Gene Set Enrichment Analysis (GSEA) implemented in WebGestalt [46,47].

### 2.6. Inferential EMT Metric

The epithelial-mesenchymal transition (EMT) metric previously described [48,49] was applied to transcriptomic datasets above to calculate the extent of ‘EMT-ness’ on a scale of 0 (fully epithelial) to 2 (fully mesenchymal). This inferential metric considers a set of EMT-relevant predictor transcripts and a cross-platform normalizer transcript set and uses them to probabilistically categorize samples into epithelial, mesenchymal, or hybrid E/M. For each sample i, an ordered triple Si = (PE, PE/M, PM) characterizes the probability of belonging to epithelial, hybrid E/M, or mesenchymal phenotype. These probabilities are then projected onto the range (0–2): epithelial samples get placed close to 0 and mesenchymal samples close to 2, whereas maximally hybrid E/M samples are assigned values close to 1.

### 2.7. Patient Survival Analysis

The Kaplan-Meier Plotter online web tool was used to assess the association of the gene signature consisting of upregulated genes in CTCcl+ TNBC PDXs and RFS in 360 basal-like BC patients, most of whom had TNBC [50,51,52]. Fifty-four upregulated genes with *p* < 0.01 in CTCcl+ tumors were entered into the database to obtain Kaplan-Meier survival plots, hazard ratios with 95% confidence intervals, and log-rank *p*-values. The “JetSet best probe set” option was selected to select the optimal probe set for each gene [53].

## 3. Results

### 3.1. Protein Profiling of TNBC PDX Models

Available RPPA quantitative data from six CTCcl− and three CTCcl+ TNBC PDX tumors were used to investigate expression levels of 171 total and phosphorylated proteins that belong to several cellular processes and proteins, including EMT, DNA-damage, apoptosis, proliferation and cell cycle, growth factor receptors, nuclear receptors/transcriptional regulators, and metabolic enzymes [40]. The analysis of differentially expressed proteins (Supplementary Table S1) revealed higher levels of the anti-apoptotic protein Bcl2 (FDR *q*-value < 0.0001, fold-change (FC) 3.5) in CTCcl+ TNBC PDX tumors (Table 1). Additionally, the fatty acid metabolism regulator ACC1 was found to be the only significantly downregulated protein (FDR *q*-value = 0.0005, FC 0.3) in CTCcl+ tumors compared to CTCcl− TNBC PDXs (Table 1).

### 3.2. Bcl2 Expression in TNBC PDX Tumors with and without CTCcls

**Figure 2:**
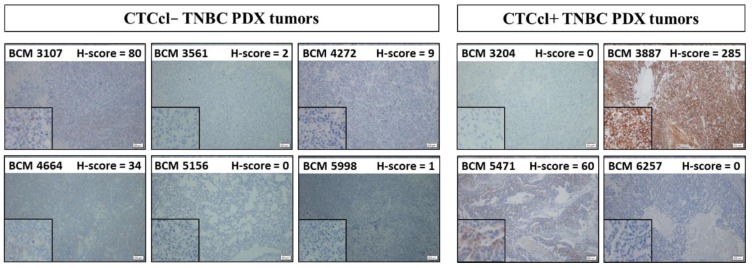
IHC analysis of B-cell lymphoma 2 (Bcl2) expression with H-score in CTCcl− vs. CTCcl+ triple-negative breast cancer (TNBC) PDX tumors. Primary tumor tissues were sectioned and stained individually with an anti-Bcl2 antibody after antigen retrieval. Sections were exposed with metal enhanced 3,3′-Diaminobenzidine (DAB) substrate and counter-stained with hematoxylin. Histoscore was assigned based on staining intensity (0 (absent), 1+ (weak), 2+ (moderate), or 3+ (strong)) and the percentage of cells stained.

We examined the expression of Bcl2 in primary tumors from all 10 TNBC PDX models by IHC staining. A median H-score of 34 for Bcl2 expression in TNBC PDX tumors was considered as a cut-off for analysis. A similar scoring system for Bcl2 has been recently reported by a few other studies [54,55]. Only one out of six CTCcl− TNBC PDX tumors had an H-score of > 34 (BCM-3107 with H-score of 80). On the other hand, two out of four CTCcl+ TNBC PDX tumors had an H-score of > 34 for Bcl2 (BCM-5471 with an H-score of 60; BCM-3887 with an H-score of 285) (Figure 2). Although an optimal cut-off for Bcl2 score for positivity requires further research, a dichotomized median H-score as cut-off was used here based on its commonality in several other studies [56,57,58,59].

### 3.3. Gene Signature Associated with CTCcl-Specificity

**Figure 3:**
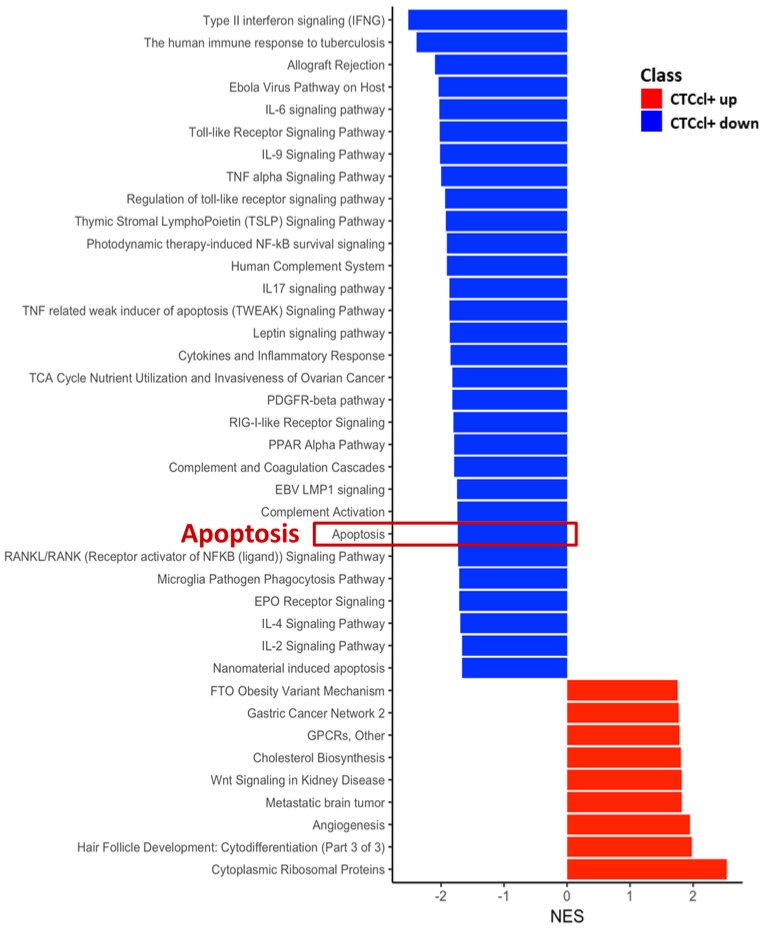
Pathway analysis showing apoptosis as one of the downregulated pathways in CTCcl+ samples. A total of 39 enriched pathway terms were identified with *p*-value < 0.05 and a false discovery rate (FDR) *q*-value < 0.1, and the enrichment score was given as normalized enrichment score (NES).

To identify genes associated with a higher propensity of generating CTCcls and consequently with a higher metastatic potential, we conducted a genome-wide transcriptomic analysis of six CTCcl− and four CTCcl+ TNBC PDX models. None of the genes in the RNA-Seq analysis had FDR < 0.05 (Supplementary Table S2). We next performed GSEA to elucidate potential biological processes that were associated with the generation of CTCcls. The normalized gene counts consisted of 12,778 features (merged the redundant features and filtered for no variance). The differential analysis through edgeR resulted in log2-FC and associated *p*-value and FDR *q*-value. We used the log2-FC as input for GSEA-based enrichment for biological pathways from Wikipathway [45]. We identified 39 enriched pathway terms with *p*-value < 0.05 and FDR *q*-value < 0.1, and the enrichment score was given as normalized enrichment score (NES). Pathway terms, such as ‘cytoplasmic ribosomal proteins (NES = 2.53)’, ‘angiogenesis (NES = 1.95)’, ‘metastatic brain tumor (NES = 1.82)’, ‘cholesterol biosynthesis (NES = 1.8)’, ‘G protein-coupled receptors (GPCRs) (NES = 1.79)’ were among the top-enriched pathways positively associated with CTCcls+ TNBC PDX tumors (Figure 3, Supplementary Table S3). Interestingly, our analysis also revealed that genes within GSEA terms relating to ‘type II interferon signaling (NES = −2.52)’, ‘antigen processing and presentation (NES = −2.38)’, ‘tumor necrosis factor (TNF) signaling pathway (NES = −2.04)’, and ‘apoptosis (NES = −1.73)’ were downregulated in CTCcl+ TNBC PDX models (Figure 3, Supplementary Table S3).

### 3.4. Expression of Plakoglobin, CD44, and PAK2 in TNBC PDX Tumors

We also examined the expression levels of *JUP* (gene for plakoglobin), *CD44*, and *PAK2* in CTCcl+ vs. CTCcl− TNBC PDX models as they were reported to have a role in tumor cell aggregation/CTCcl formation and subsequent metastasis [8,27]. None of the three genes were differentially regulated in our analysis even with FDR cut-off of 0.1: *PAK2* (log2-FC = 0.88, *p*-value < 0.05); *JUP* (log2-FC = −0.54, *p* = 0.29); and *CD44* (log2-FC = −0.93, *p* = 0.17).

### 3.5. Prediction of Inferential EMT Metric for TNBC PDX Models

**Figure 4:**
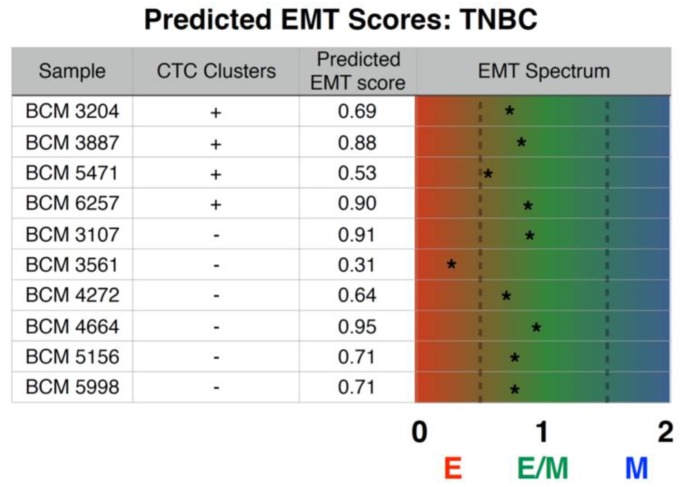
Inferential epithelial-mesenchymal transition (EMT) metric assigned to TNBC PDX models. This metric considers a set of EMT-relevant predictor transcripts and a cross-platform normalizer transcript set and uses it to probabilistically categorize samples into epithelial (close to 0), mesenchymal (close to 2), or hybrid E/M (close to 1).

EMT has been proposed to play an important role in promoting CTC formation in epithelial cancers by increasing tumor cell invasiveness, intravasation into blood vessels, and survival in the peripheral system. However, recent studies have argued that EMT is not an ‘all-or-none’ process as initially postulated, but cells may stably acquire one or more hybrid epithelial/mesenchymal phenotype(s) [60]. To correlate differences in the EMT status of TNBC PDX tumors with the presence and absence of CTCcls, we applied our recently developed inferential EMT metric to calculate EMT scores for each PDX tumor. This metric uses canonical epithelial and mesenchymal markers and computes an ‘EMT score’ on a scale of 0 (fully epithelial) to 2 (fully mesenchymal) [48,49]. These analyses indicated that the extent of EMT activated in TNBC PDX tumors generating CTCcls was not significantly different from those tumors where no CTCcls were detected (Figure 4).

### 3.6. Prognostic Values of CTCcl-Associated Signature in Basal-Like BC Patients

**Figure 5:**
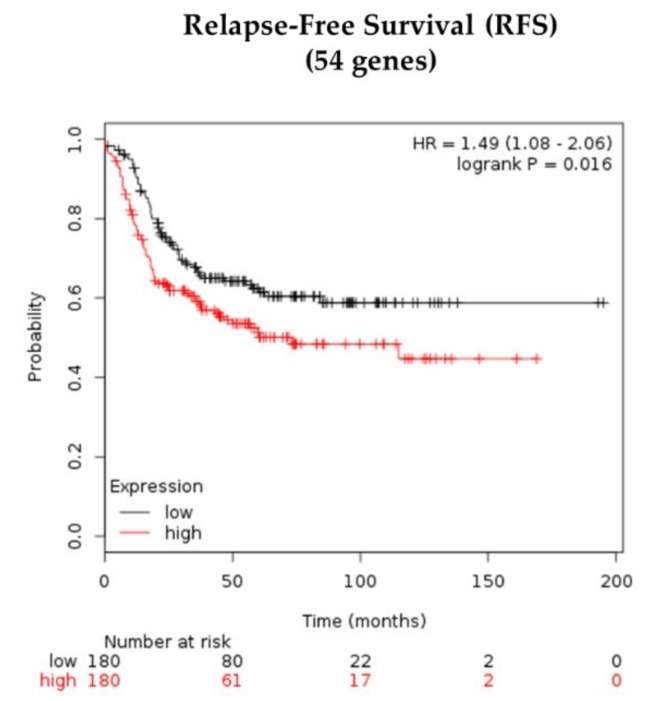
Prognostic value of CTCcl-associated gene signature in patients with basal-like breast cancer (BC), most of whom have TNBC. Fifty-four genes were found to be upregulated in TNBC PDX models with CTCcls. Gene-expression profiles of 360 basal-like BC were each scored with this signature. Kaplan-Meier curve compares distant metastasis-free survival in BC patients with relatively higher signature scoring versus those with lower scoring. Patient data were extracted from publicly available datasets using http://kmplot.com/.

A gene signature associated with CTCcl positivity was defined by selecting the 54 upregulated genes (Supplementary Table S4) with a log2-FC of 0.9 and *p*-value < 0.01 in CTCcl+ vs. CTCcl− TNBC PDX tumors. The CTCcl positivity signature was significantly associated with poor RFS (Hazard ratio (HR) 1.49, 95% confidence interval (CI) 1.08–2.06, *p*-value = 0.016) in a publicly available cohort of 360 patients with basal-like BC (http://kmplot.com/) (Figure 5).

## 4. Discussion

Tumor cells in the circulation were once regarded as sporadic events with a lack of tools and techniques to detect and identify them in cancer patients [61]. However, with recent technological advancements, the CTCs are now well established as prognosis predictors of survival and treatment outcomes in metastatic cancers [5,6,7]. CTCcls represent a unique subset of CTCs with a greater survival advantage, higher metastatic potential, and resistance to chemotherapy [8,9,10]. The clinical prognostic value of CTCcls at baseline and after treatment for predicting progression-free survival and overall survival in cancer patients has also been recently demonstrated [16,17,18,19,20,21]. While the prognostic value of CTCcls has been shown primarily in metastatic cancer patients, it is unknown whether the presence of CTCcls in locally-advanced or early-stage disease would predict early metastatic recurrence. Likewise, the tumor characteristics that give rise to CTCcls and the pathophysiological events by which CTCcls originate are also largely unknown [22]. Additional clinical studies are required to fill the knowledge gap.

Our RPPA and transcriptomic analyses identified high Bcl2 expression and downregulation of apoptosis pathways associated with CTCcls. Bcl2 is an oncogene that promotes survival and regulates pro- and anti-apoptotic pathways [62,63], which is one of the hallmarks of cancer [64]. Approximately 75% of breast tumors are positive for Bcl2 [65,66]. While Bcl2 is a favorable prognostic marker in estrogen receptor-positive BC and an independent predictor of clinical outcome in patients treated with endocrine therapy, its expression is a poor prognostic factor in TNBC patients, especially in the absence of adjuvant therapy [67]. In addition, Bcl2 is also reported as an independent prognostic marker [68] and is associated with poor RFS [69] and worse overall survival [70] in TNBC patients receiving anthracycline-based adjuvant chemotherapy. Although highly expressed in 20–40% of TNBC/basal-like BC [68,69,71,72], Bcl2 has not been clinically investigated as a drug target in TNBC patients. In the preclinical setting, venetoclax (ABT-199), a Food and Drug Administration (FDA)-approved anti-Bcl2 drug, was showed to increase anti-tumor effects of doxorubicin on subcutaneously injected MDA-MB-231 cells in xenograft models [73]. Similarly, ABT-737, an inhibitor of Bcl2 and Bcl-xl, sensitized TNBC PDXs to docetaxel [74] and accelerated apoptosis in irradiated TNBC cells in vitro and in vivo [74] in another study. Further preclinical and clinical validation of Bcl2 as a predictor and driver of CTCcls formation and metastasis is warranted in TNBC. Besides apoptosis, tumor microenvironment/cytokine-specific signature and tumor cell metabolism-related pathways are the two other prominent pathways that are downregulated in our enrichment analysis. However, the PDX tumors are maintained in SCID/Beige mice, which is severely immunocompromised and lack both T-cells and B-cells and have diminished natural killer (NK) cell activity. How these pathways play a role in CTCcls formation and maintenance in PDXs, needs further investigation.

The uniqueness of our study comes from the use of PDX models to investigate CTCcls. While many preclinical CTC studies are using cell line xenograft models, we have pioneered the characterization of various BC PDX models for the presence of CTCs, CTCcls, and disseminated tumor cells (DTCs) in bone marrow [24]. PDX models are clinically relevant and phenotypically stable because they maintain morphologic and structural characteristics and also metastasize as original tumors [75,76]. CTCs/CTCcls are extremely rare in patients when only 5–10 mL of blood can be withdrawn from patients at a time. In contrast, at least 1/4th to 1/3rd of the whole blood volume can be collected from a mouse for detecting CTCcls [38]. Moreover, CTCs/CTCcls from PDX models can be interrogated in their unperturbed state, in the absence of treatment, to understand their influence on metastatic processes. BC PDXs also recapitulate many key biological aspects of the original patients’ tumor, including molecular heterogeneity and drug sensitivity, and hence are considered excellent models of translational research [77,78]. In our study, we utilized the existing RPPA and transcriptomic (RNA-Seq) data of PDX tumors, performed using total protein and RNA samples, respectively. While we cannot rule out the contribution from mouse stromal cells, PDX tumors cluster according to their original PAM50 subtype, and most proteins included in the RPPA panel are tumor-specific. For Bcl2 IHC assay, we used an anti-human Bcl2 antibody that does not cross-react with mouse protein, and the IHC scoring agreed with the signal from RPPA analysis. The ongoing bioinformatic effort for computationally editing the stromal reads may help in the future analysis of PDX tumors.

Single-cell RNA sequencing of clustered versus single CTCs has identified plakoglobin, a cell adhesion protein, as a highly expressed gene in CTCcls [8]. High plakoglobin expression was also found on primary tumors of BC patients and served as an independent prognostic factor for distant-metastasis-free survival (DMFS) in BC patients undergoing neoadjuvant chemotherapy [8,26]. In a recent study, intercellular CD44 homophilic interactions and its subsequent interactions with PAK2 were reported to mediate tumor cell aggregation and polyclonal metastasis in BC PDX models [27]. In our study, we took a different approach of interrogating protein and transcriptomic data from CTCcl+ versus CTCcl− TNBC PDX tumors to identify global molecular pathways responsible for CTCcls and to identify ‘druggable’ targets for combating CTCcl-driven metastasis. While we identified Bcl2, a druggable target, for future interrogation and validation, the evaluation of a larger panel of TNBC PDX models and patients is necessary to identify additional biomarkers and targets.

## 5. Conclusions

Our study shows for the first time that OMICS analysis of PDX tumors can determine CTCcl-specific protein markers and gene signature to predict outcomes in patients. Primary tumors with active anti-apoptotic and/or survival pathways may promote CTCcls formation and increase the risk of distant metastasis. Considering the molecular heterogeneity within TNBC, a large panel of TNBC PDX needs to be characterized to ensure robust analysis with high precision. Discovering tumoral predictors and drivers of CTCcls that can serve as biomarkers may identify patients at higher risk of metastatic relapse. Understanding the biology behind the genesis of CTCcls can also elucidate pathways that can be targeted using novel therapies for the prevention and treatment of CTCcl-associated metastasis in TNBC.

## Figures and Tables

**Table 1:** Differentially expressed proteins in TNBC PDX tumors with versus without CTCcls based on RPPA analysis.

Antibody	Average Log2 Intensity on TNBC-PDX Tumors		*p*-Value	FDR *q*-Value	Expression Levels	FC
	CTCcl+	CTCcl−				
* Bcl2	1.63	−0.18	<0.0001	<0.0001	↑	3.5
^#^ ACC1	−1.55	0.02	<0.0001	0.0005	↓	0.3

*, significantly upregulated protein; ^#^, significantly downregulated protein. Abbreviations: ACC1, acetyl CoA carboxylase-1; Bcl2, B-cell lymphoma 2; CTCcl, circulating tumor cell cluster; FC, fold change; FDR, false discovery rate; PDX, patient-derived xenograft; TNBC, triple-negative breast cancer; RPPA, reverse-phase protein array.

## Supplementary Materials

The following are available online at https://www.mdpi.com/2077-0383/8/11/1772/s1. Table S1: Differential expression of proteins and phosphoproteins by RPPA analysis in CTCcl+ vs. CTCcl− TNBC PDX models; Table S2: Differentially expressed genes by RNA-Seq analysis in CTCcl+ vs. CTCcl− TNBC PDX models; Table S3: Differentially regulated biological pathways elucidated by GSEA in CTCcl+ vs. CTCcl− TNBC PDX models; Table S4: List of 54 upregulated genes with *p*-value < 0.01 in TNBC PDX models associated with the CTCcl-specific signature.
